# RF phase modulation improves quantitative transient state sequences under constrained conditions

**DOI:** 10.1007/s10334-025-01293-9

**Published:** 2025-09-11

**Authors:** Miha Fuderer, Hongyan Liu, Oscar van der Heide, Cornelis A. T. van den Berg, Alessandro Sbrizzi

**Affiliations:** Computational Imaging Group for MR Diagnostics & Therapy, Center for Image Sciences, University Medical Center Utrecht, Heidelberglaan 100, 3585CX Utrecht, The Netherlands

**Keywords:** Multiparametric magnetic resonance imaging, MR-STAT, Quantitative MRI, RF phase modulation, Sequence optimization

## Abstract

**Objective:**

Within gradient-spoiled transient-state MR sequences like Magnetic Resonance Fingerprinting or Magnetic Resonance Spin TomogrAphy in Time-domain (MR-STAT), it is examined whether an optimized RF phase modulation can help to improve the precision of the resulting relaxometry maps.

**Methods:**

Using a Cramer-Rao based method called BLAKJac, optimized sequences of RF pulses have been generated for two scenarios (amplitude-only modulation and amplitude + phase modulation) and for several conditions. These sequences have been tested on a phantom, a healthy human brain and a healthy human leg, to reconstruct parametric maps (${T}_{1}$ and ${T}_{2}$) as well as their standard deviations.

**Results:**

The amplitude + phase modulation scenario systematically resulted in lower noise levels than the amplitude-only modulation scenario. On average, the difference was around 34%, but it was substantially larger for scans acquired under SAR restrictions. Compared to amplitude-only, in the amplitude + phase modulation scenario, the relevance of an inversion pulse and of a pause were greatly reduced, at least considering overall precision and in-phantom accuracy.

**Conclusion:**

The application of an optimized RF phase modulation in quantitative transient-states MRI is beneficial for almost all tested scenarios and conditions, in particular under SAR restrictions Furthermore, RF phase modulation reduces the need for inversions pulses and pauses.

**Supplementary Information:**

The online version contains supplementary material available at 10.1007/s10334-025-01293-9.

## Introduction

In MR Imaging, most diagnostic tasks are still based on qualitative images, (e.g. ${T}_{1}$ or ${T}_{2}$-weighted images). Next to qualitative imaging, quantitative imaging [1]—e.g. the mapping of ${T}_{1}$ or ${T}_{2}$-values over a multiplicity of voxels—has also been considered throughout the history of MR. More recently, techniques that allow *simultaneous* estimation of ${T}_{1}$ and ${T}_{2}$ (and potentially other parameter-maps) have been developed. These can broadly been categorized as steady-state methods (e.g., DESPOT/JSR [2], DESS [3], PLANET [4], QRAPTEST [5], QRAPMASTER [6], STAGE [7], QALAS [8], MR-Multitasking [9], EPTI) and transient-state methods. The latter include MR Fingerprinting (MRF) [10], Magnetic Resonance Spin TomogrAphy in Time-domain (MR-STAT) [11], Quantitative Transient-state Imaging [12] and Hybrid-state imaging [13]. In these transient-state methods, RF pulses are usually time-varying over their sequence; consequently, the magnetization is continuously in a transient-state. This RF-pulse variability gives many degrees of freedom to optimize a sequence of RF pulses with respect to ${T}_{1}$ and ${T}_{2}$ encoding, which is a non-trivial task. Many optimization approaches are limited to the *amplitude* of RF pulses [12, 14–25], while it has recently been shown that appropriate application of phase is beneficial [13, 26–29]. Interestingly, almost all of these methods [10–16, 18, 22, 23, 27, 29–31] apply an initial inversion pulse. This contributes to ${T}_{1}$-encoding. However, the initial inversion pulse brings also additional power deposition, model imperfections of its own [32] and a need for (partial) magnetization-relaxation before the pulse [33]—which is relevant if the sequence requires repetition, as in 3D [34, 35] or time-resolved [36] scanning, or whenever the number of repetitions for one single volume substantially exceeds 1000, as in Flow-MRF [37].

In this work, we examine the benefit of simultaneously optimizing both amplitude and phase modulation of the RF pulse sequence over optimizing the amplitude only. In particular, we analyze the impact of phase modulations on other sequence design choices such as the insertion of inversion pulses and/or pauses. We do this in the framework of MR-STAT, which uses a Cartesian gradient-spoiled gradient-echo sequence with varying RF excitation pulses and typically applies 5 or 6 repetitions of all required phase-encoding values. Each readout has “seen” a different history of RF-pulses and therefor a different sensitivity to relaxation properties; also, each readout has undergone a specific phase-encoding. No explicit Fourier Transform is performed, but via a large-scale non-linear inversion process, spatial relaxation maps are estimated.

For our purpose, we experiment on sequences where scan segments are possibly repeated; each segment consists of a set of excitation pulses, whereby this set may be preceded by a pause and/or an inversion pulse. A relevant acquisition strategy is contiguous scanning, involving repetitions of segments without interleaved pauses or inversion pulses [33–37]. Finally, reducing the RF power deposition is of particular interest for high-field applications.

The aforementioned acquisition strategies can be applied with additional RF phase modulation. For example, Wang [27], Boyacioglu [28] and Liu [29] apply small, constant quadratic RF-phase increments of ±2°$/{T}_{R}^{2}$. These small quadratic phase increments are somewhat reminiscent of RF-spoiling [38] and its application in MRF [39, 40]; however, RF-spoiling typically applies much higher 2nd derivatives, which are designed to minimize the contribution of stimulated echoes; typically RF-spoiling uses the golden angle, 137.5°, or an angle of 117° as derived by Zur et al. [38]. In our work, we use very small quadratic increments [27–29] and go one step further by numerically *optimizing* them as a time-dependent function. For this purpose, we apply BLock Analysis of a K-space-domain Jacobian (BLAKJac) [24].

By means of phantom scans, and in vivo brain and knee measurements, we examine the effect on precision of optimized RF phase modulation and of pauses [33] and inversion pulses. The necessity (or absence thereof) of pauses and inversion pulses is deemed relevant in the design of contiguous, time efficient cyclic sequences, which is useful when designing 3D or time-resolved scanning in both MR-STAT and MRF.

## Methods

### Sequence type

In our experiments, we used a non-balanced Cartesian MR-STAT sequence. Here, “non-balanced” implies a constant nonzero gradient area between any two successive RF-pulses. The area corresponds to 1 cycle per voxel, which in our case is $2\pi$/mm (in rad). The scan was applied on a 3T scanner (Philips Elition), with ${T}_{R}$ = 10 ms, ${T}_{E}$ = 5 ms, voxel size 1 mm × 1 mm, slice thickness of 5 mm, with a field of view of 224 mm by 224 mm, requiring 224 phase-encoding steps. The set of phase-encoding steps was repeated 6 times, allowing MR-STAT reconstruction of maps of proton density, ${T}_{1}$ and ${T}_{2}$. In total, $224\times 6=1344$ readout-lines were acquired for a total acquisition length of 13.44 s.

### Generation of RF pulse sequences

RF pulse sequences were generated using BLAKJac [24]. BLAKJac is a framework that allows to simultaneously optimize amplitude *and phase* modulation of transient-state sequences. BLAKJac is, in essence, a Cramer-Rao based technique, whereby the noise level in the output relates to the noise level in the input via the matrix ${\left({{\boldsymbol{J}}}^{H}{\boldsymbol{J}}\right)}^{-1}$, where ${\boldsymbol{J}}$ is the Jacobian of the signal model, i.e., the expected signal as a function of tissue properties; this is derived using Extended Phase Graphs [41]. In an iterative process, sequence properties are optimized to minimize output noise. See also Supporting Information 1. Thereby, BLAKJac generates an estimate of the noise level in the reconstructed maps, given an RF pulse sequence and the corresponding gradient encoding sequence. This approach allows for fast optimization of RF pulse sequences, while taking into account the gradient encoding strategy.

The RF sequence optimization was performed for two scenarios:

- Amplitude-only modulation: the phase of the RF pulses was fixed to zero; the real part (which was allowed to be negative) was parameterized as a cubic-spline interpolation based on 6 equidistant points in time. The actual object of the optimizer were the values (in this case, the RF amplitudes) at these 6 points.
- Amplitude + Phase modulation: the following variables were optimized simultaneously: (1) the “amplitude” (which was represented by a real number, allowed to be negative) and (2) ${\phi }^{{\prime}{\prime}}$, a variable representing the second derivative of the phase. Both time-dependent functions were represented by cubic-spline expansions (see scenario A). To derive the phase at each RF pulse, we numerically integrated $\phi {\prime}{\prime}$ twice: ${\phi }_{k}={\sum }_{j=1}^{k}({\sum }_{i=1}^{j}{\phi }_{i}^{{\prime}{\prime}})$. Note that a constant value of ${\phi }^{{\prime}{\prime}}$ would lead to a phase ${\phi }_{k}$ that is quadratic in $k$; a very smoothly varying $\phi {\prime}{\prime}$ is comparable to a locally quadratic phase (locally, in time), whereby the quadratic component slowly changes over time.

**Fig. 1:**
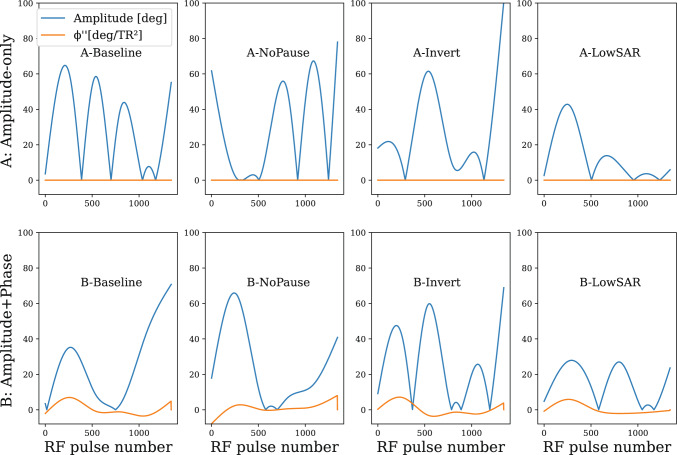
Optimized RF pulse sequences for the scenarios of Amplitude-only modulation (top row) and Amplitude + Phase modulation (bottom row). Absolute value of the RF excitation angles in blue (note: it is the absolute value of a real function that is allowed to be negative); the RF phase (orange) is displayed in terms of its second order derivative (degrees per ${T}_{R}^{2}$)

For each of the scenarios described above (i.e. Amplitude-only and Amplitude + Phase), RF pulses were optimized for four different conditions, as specified in Table 1. This resulted in 8 combinations of scenarios and conditions (see Fig. 1).

**Table 1:** The characteristic of the four conditions. In each condition, the sequence consisted of segments of 1344 repetitions (TR = 10 ms) of RF excitations with interleaved readouts

Condition name	Inversion pulse	Pause	SAR restrictions (max RMS flip angle [°])
Baseline	No	5 s	40
No-pause	No	0	40
Inversion	Yes	5 s	40
Low Sar	No	5 s	10 or 20

Except for the No-pause condition, each segment was preceded (or followed) by a pause (as suggested in previous work [33, 56]). In the No-pause condition, the first pulse of the sequence followed 10 ms after the last pulse of the previous one, and a dummy sequence was applied up-front to ensure steady-state conditions. In the Inversion-condition, each segment is preceded by an adiabatic inversion pulse

For all 8 combinations of scenarios and conditions, the optimization was aimed at minimizing the maximum relative noise level in the ${T}_{1}$ and ${T}_{2}$ maps.

For further details on the optimization method, see Ref. [24] (software resources available [42]) as well as supplementary material 1.

### Measurements

Each combination of scenario and condition was experimentally validated. To estimate the standard deviations in the resulting parameter maps, each sequence was re-scanned and reconstructed 10 times. The reconstruction took into account the knowledge of the ${B}_{1}^{+}$ transmit field, which was pre-acquired using the dual refocusing echo acquisition mode [43]. The ${B}_{1}^{+}$-map was acquired once per anatomy-type (see below).

The aforementioned set of $2\times 4\times 10$ scans was applied on a Eurospin II phantom set [44], on a brain and on a knee of a healthy human volunteer (with approved consent according to the guidelines of the ethics committee).

### Evaluation

**Fig. 2:**
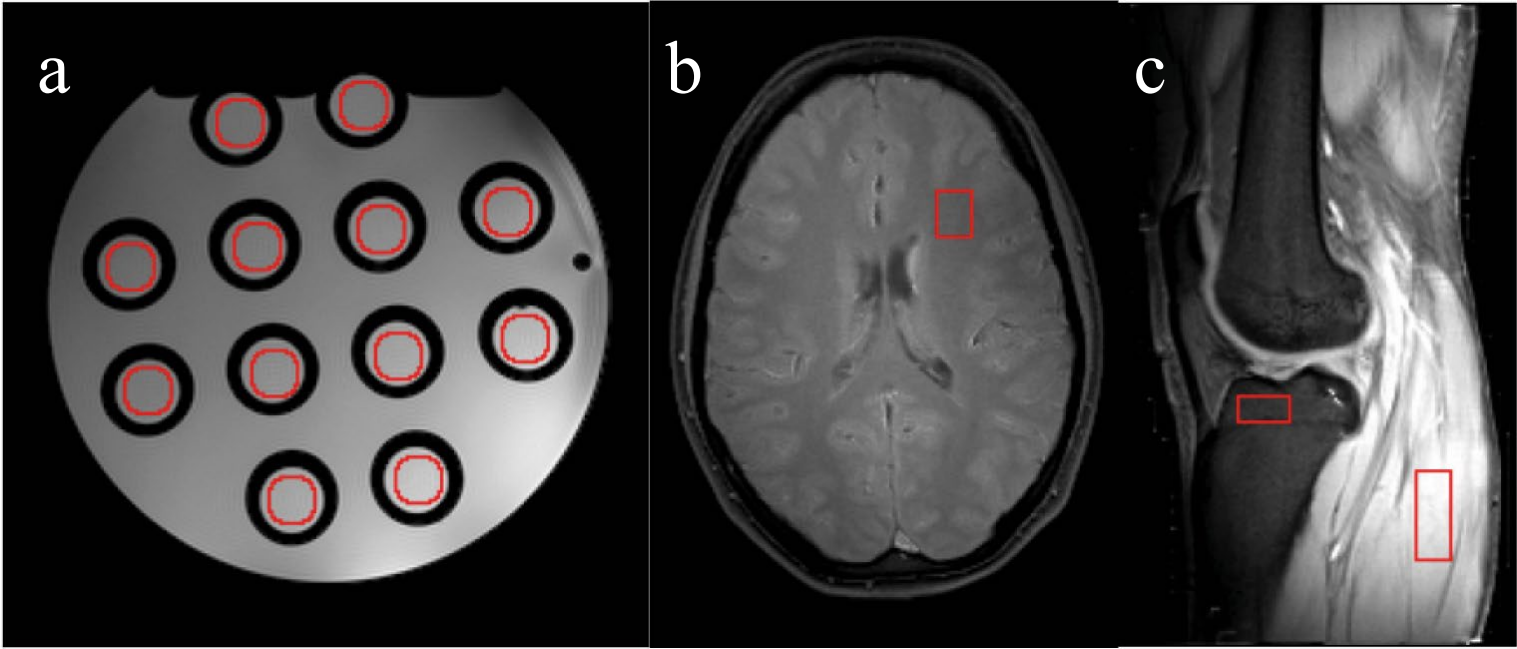
Placement of the regions of interest: **a** 12 round ROIs on the vials of the Eurospin phantom; **b** on the white matter of the brain and **c** on muscle and on bone marrow in the lower leg. (Shown images are proton-density maps.)

In the phantom, a ROI was defined on each of the 12 vials (Fig. 2a). Each of these ROIs had an area of 193 pixels. We thus obtained a multiplicity of 12 vial-dependent standard deviations. For more concise reporting of statistics, we subsequently calculated the average standard deviation as a mean over the 12 standard deviations of the individual vials. The noise standard deviation was estimated as $\sigma =\frac{1}{10}{\sum }_{i=1}^{10}\underset{{\boldsymbol{r}}\in {\mathrm{ROI}}}{\mathrm{std}}({m}_{i}\left(r\right)-\frac{1}{10}{\sum }_{j=1}^{10}{m}_{j}\left(r\right))$, where ${m}_{i}$ is the $i$-th reconstruction of the ${T}_{1}$ map or the ${T}_{2}$ map.

In the brain scan, we defined a ROI in the white matter, since this tissue allows for the clear definition of a ROI over a relatively large area. In our case, the ROI contained $15\times 12$ pixels (Fig. 2b). Similarly, in the knee image, ROIs were defined in the muscle (size $30\times 11$ pixels) and in the red marrow (tibia, plateau epiphysis, ROI area of $8\times 17$ pixels). See Fig. 2c. Since there was no observable macroscopic motion, the ROI position was kept constant over the 10 re-scans.

In the volunteer data, $\sigma$ was estimated in the same way as in the phantom. Yet, as motivated in the Discussion, this value was divided by the mean relaxation value over the ROI (i.e., by $\mu =\frac{1}{10}{\sum }_{i=1}^{10}\underset{{\boldsymbol{r}}\in {\mathrm{ROI}}}{\mathrm{mean}}({m}_{i}\left(r\right))$); the precision is then defined as $\mu /\sigma$. In this way, precision reflects the absence of variation of the result over test/retest.

When comparing sequences, we calculate the metric of *precision gain*. For each parameter, tissue type and condition, the precision gain was calculated by taking the logarithmic ratios [45, 46] of precision values, $\left(\mathrm{ln}\left(\frac{{\mu }_{B}/{\sigma }_{B}}{{\mu }_{A}/{\sigma }_{A}}\right)\right)\cdot 100\%$. By averaging these gains over the two relaxation parameters (${T}_{1}$ and ${T}_{2}$), and over four tissue-types, we obtain rows (b) through (e) of Table 2, with their average reflected in row (a).

**Table 2:** Overview of measured relative precision levels

Row label	Description		Gain
(a)	Average precision gain using phase		33.9%
(b)	Precision gain of phase without inversion		40.3%
(c)	Precision gain of phase without inversion or pause		29.9%
(d)	Precision gain of phase with inversion		16.9%
(e)	Precision gain of phase in SAR-restricted situation		48.5%
(f)	Precision gain of inversion pulse	Amplitude-only	32.5%
		Amplitude + Phase	9.1%
(g)	Precision gain of pause	Amplitude-only	9.7%
		Amplitude + Phase	20.1%
(h)	Precision-efficiency gain of pause	Amplitude-only	−6.1%
		Amplitude + Phase	4.3%

(a) Average precision gain by applying Amplitude + Phase modulation over Amplitude-only modulation, averaged over the two relaxation parameters, over substances (phantom, white matter, muscle, marrow) and over conditions. (b)–(e) Gain for the specific conditions. (f) Shows the gain realized by adding inversion pulses. We observe an precision gain from the inversion pulses, but this benefit is very low if the sequence is optimized on Amplitude + Phase modulation. (g) The gain by adding pauses, which suggests that pauses increase precision by 10 to 20%. Yet, when calculating precision-efficiency by taking into account the increased scan time, the net gain by adding pauses is practically zero (h)

The means of the phantom vials do allow, in principle, for a quantitative analysis on accuracy. Gold-standard ${T}_{1}$ values of the vials were established by a multiplicity of inversion-recovery measurements with differing inversion times, while ${T}_{2}$ values were established using a multiplicity of single-echo spin-echo measurements. This results into values ${g}_{vn}$, where vial $v\in [1\dots 12]$ and relaxation map ${T}_{n}$, $n\in \{\mathrm{1,2}\}$.

For the four conditions and the two scenarios outlined in the main document, mean values were taken over Regions of Interest located on each of the 12 phantom vials. This resulted in $2\times 4\times 12\times 2$ figures ${\mu }_{csvn}$, representing the mean (over the vial voxels and over the 10 re-scans) for condition $c$, scenario $s\in \{A,B\}$, vial $v$ and relaxation map ${T}_{n}$.

## Results

**Fig. 3:**
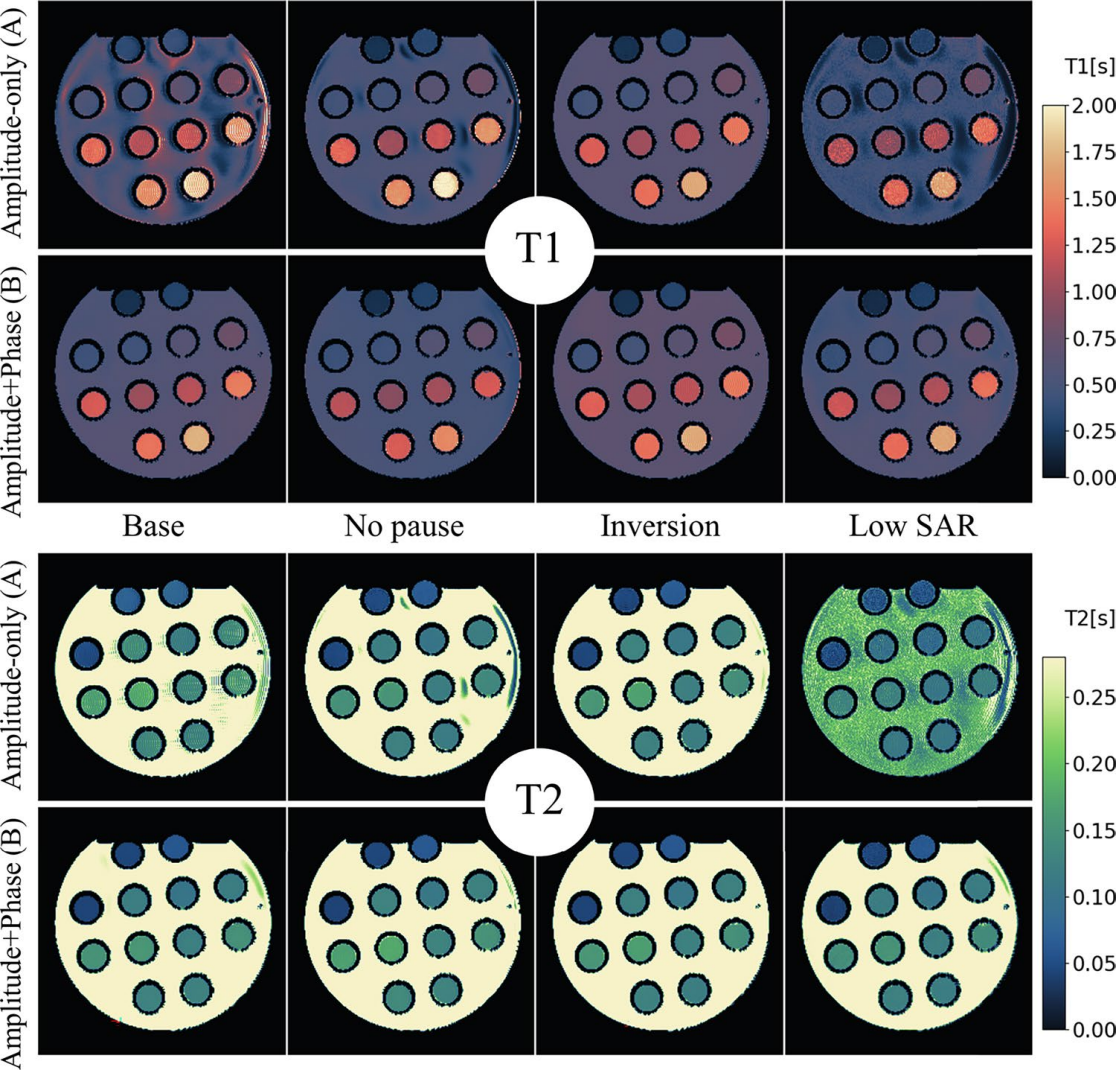
${T}_{1}$ maps and ${T}_{2}$ maps of the 8 combinations of scenario and condition in the Eurospin phantom. the background liquid may show some flow artifacts, which are irrelevant for our discourse. The twelve vials are used for further analysis

**Fig. 4:**
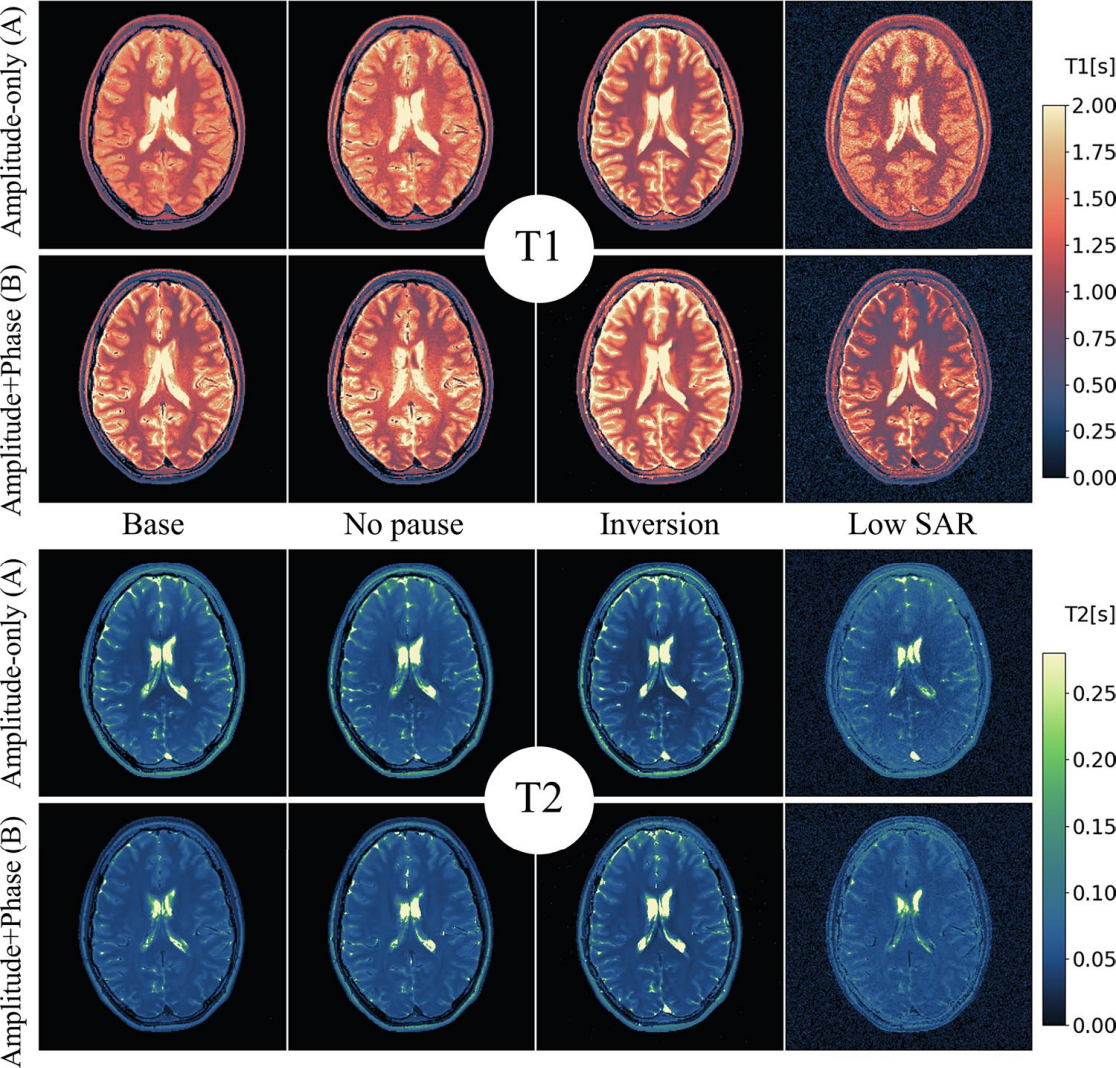
${T}_{1}$ maps and ${T}_{2}$ maps of the 8 combinations of scenario and condition in the brain of a healthy volunteer. (Phase-encoding direction: LR)

**Fig. 5:**
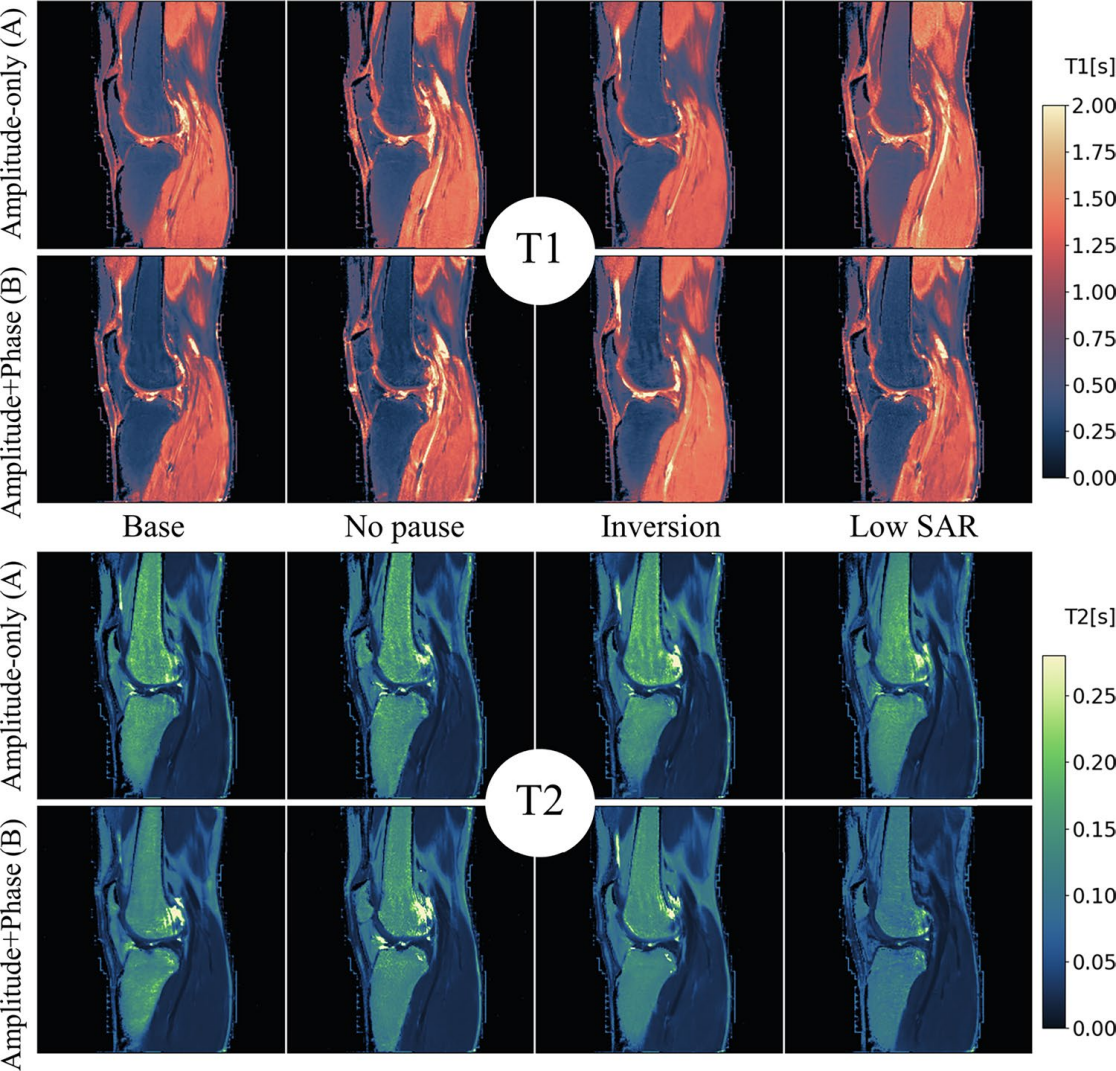
${T}_{1}$ maps and ${T}_{2}$ maps of the 8 combinations of scenario and condition in the knee of a healthy volunteer. (Phase-encoding direction: AP)

The RF pulse sequences resulting from the optimization are shown in Fig. 1. Figures 3, 4 and 5 show the resulting ${T}_{1}$ and ${T}_{2}$ maps for phantom, brain and knee, respectively (averaged over 10 reconstructions).

### Measured noise levels

**Fig. 6:**
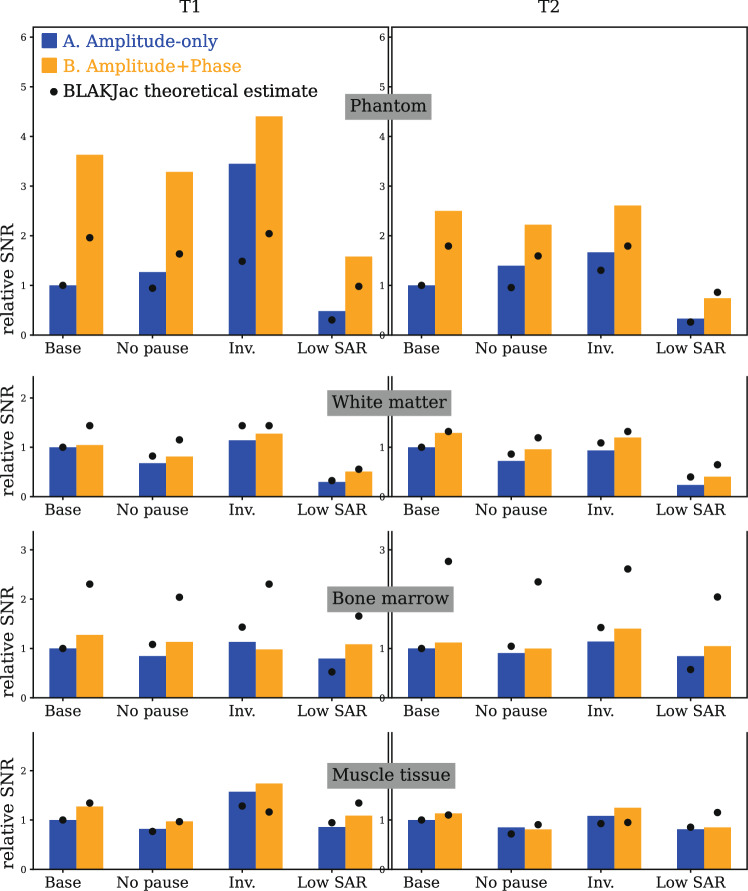
Barplots of experimentally measured relative precision levels over substances, scenarios and conditions. Left: results for ${T}_{1}$. Right: results for ${T}_{2}$. The four conditions are: (Baseline) with pause but no inversion, the others are modifications thereupon. The black dots represent the BLAKJac-estimates of the expected precision. All bars have been scaled to the level of combination A-Baseline, that is, Amplitude-only modulation without inversion pulses. What is apparent is that practically all Amplitude + Phase modulated sequences (orange bars) return higher precision than the Amplitude-only modulated sequences (blue bars)

Figure 6 shows the obtained relative precision levels in all experiments. These have all been normalized with respect to the measured noise level for combination A-Baseline (Amplitude-only). The relative precision of combination A-Baseline is thus 1.

It is immediately apparent that the relative precision obtained using Amplitude + Phase modulated sequences is always higher than the relative precision from Amplitude-only modulated sequences, except for two cases where it is slightly lower—by less than 14%. This precision gain is not surprising (after all, we provide more degrees of freedom to the optimizer), but the level of improvement, 34% in average, is noteworthy. This is shown in Table 2, in particular row (a) and rows (b–e).

Row (f) of Table 2 compares the Baseline condition and the Inversion condition. This comparison has been analysed separately for the Amplitude-only modulation scenario and for the Amplitude + Phase modulation scenario. For both scenarios, averaging over substances and over ${T}_{1}$ and ${T}_{2}$ has been applied. The bars show that the presence of regular inversion pulses leads to an increase in precision. However, this gain is very low (< 10%) if the sequence is optimized on Amplitude + Phase. Without phase modulation, the influence of the inversion pulse is substantial, > 30%.

Row (g) shows the benefit of having a pause of 5 s after each segment of 13.44 s. This suggests a precision gain (10 to 20%) when using pauses. Yet, pauses increase scan time. Considering precision *efficiency*, i.e. ${\mathrm{precision}}/\sqrt{\mathrm{scantime}}$, we have to adjust the results of row (g) by $\mathrm{ln}(\sqrt{\left(13.44{\mathrm{s}}+5{\mathrm{s}}\right)/13.44{\mathrm{s}}})$. The result is shown in row (h), suggesting practically no net benefit of pauses. This is elaborated in supplementary material 2 for varying pause lengths, which suggests that pauses are never favorable in terms of scan efficiency.

### Correspondence between BLAKJac predictions and measured noise levels

**Fig. 7:**
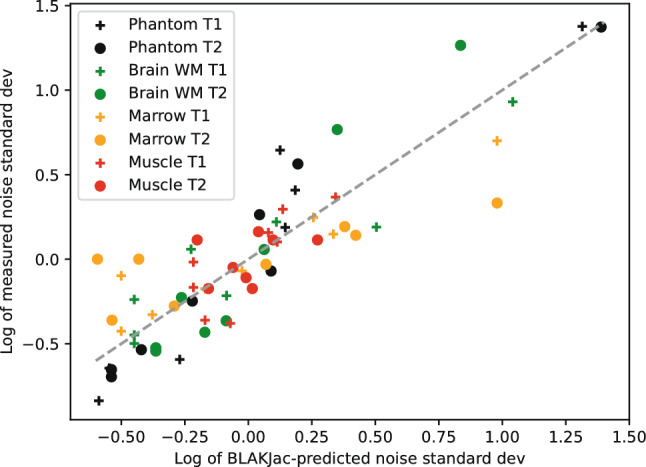
Scatter logarithmic plot of BLAKJac predicted noise (for the phantom, based on a 7-points mix of $\left({T}_{1},{T}_{2}\right)$; for tissues, the specific $\left({T}_{1},{T}_{2}\right)$ of that tissue) and measured noise levels. Within each category (combination of tissue-type and parameter-type), we have a set of 8 combinations of scenario and condition; for each set, the mean value of ln(σ) has been subtracted, where $\sigma$ indicates the standard deviation of the reconstructed or estimated noise. The dashed line indicates identity. Overall, the scatter plot shows good correlation (0.88) between prediction and measurement, although in some categories the slope is substantially lower than the expected value of 1.0; this is particularly so in marrow ${T}_{2}$ (orange bullets), where the slope is 0.33

The correspondence between BLAKJac-estimates and measurements is best expressed by the scatter plot in Fig. 7. For eight categories, where “category” is defined as a combination of tissue-type and relaxation-parameter (e.g. white matter ${T}_{2}$), we have a set of eight combinations of scenario and condition. For each combination of each category, we calculated $\mathrm{ln}({\sigma }^{B})$, where ${\sigma }^{B}$ is the noise level predicted by BLAKJac as well as $\mathrm{ln}({\sigma }^{M})$, where ${\sigma }^{M}$ is the actually measured noise level. Each category thus contains eight values of $\mathrm{ln}({\sigma }^{\mathrm{B}})$ and eight values of $\mathrm{ln}({\sigma }^{\mathrm{M}})$. Subsequently, from these, we subtracted the mean values of $\mathrm{ln}({\sigma }^{B})$ and $\mathrm{ln}({\sigma }^{M})$ per category. The resulting values, for all eight categories, were entered in Fig. 7.

The overall correlation between BLAKJac prediction and actual measurement is 0.88 with a slope of 0.94 which supports the validity of the BLAKJac analysis.

### Accuracy analysis

**Fig. 8:**
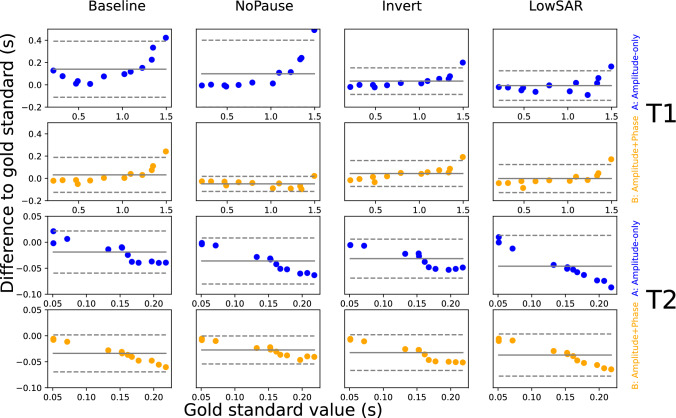
Measured inaccuracy in the phantom. It shows Bland–Altman plots for all combinations of condition and scenario. Upper eight graphs are on ${T}_{1}$, the lower eight on ${T}_{2}$

Figure 8 shows Bland–Altman plots for all combinations of condition $c$, scenario $s$ and relaxation parameter $n$ (conditions $c$ forming the columns and scenarios $s$ the rows; upper eight plots refer to ${T}_{1}$, the lower eight to ${T}_{2}$). Each graph shows the difference $({\mu }_{csvn}-{g}_{vn})$ against ${g}_{vn}$. Ideally, all $({\mu }_{csvn}-{g}_{vn})$ should be zero, the deviation from zero indicating inaccuracy.

For each value of $c$, $s$ and $n$, the mean ${M}_{csn}=\frac{1}{12}\cdot {\sum }_{v=1}^{12}({\mu }_{csvn}-{g}_{vn})$ was calculated (represented by the full grey line in the plot) as well as the corresponding standard deviation ${S}_{csn}$. The dashed lines indicate the confidence interval ${M}_{csn}\pm 1.96\cdot {S}_{csn}$.

**Fig. 9:**
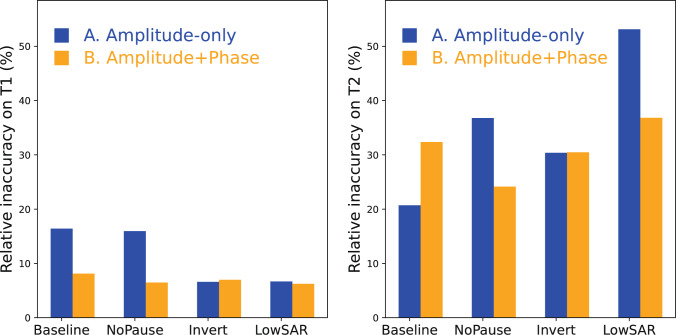
Inaccuracy values per condition, scenario and relaxation type

The data is further condensed into one single value ${\mathrm{inacc}}_{csn}$ per condition, scenario and relaxation type, by calculating ${\mathrm{inacc}}_{csn}=\sqrt{\frac{{\sum }_{v=1}^{12}{\left({\mu }_{csvn}-{g}_{vn}\right)}^{2}}{{\sum }_{v=1}^{12}{g}_{vn}^{2}}}$. These values, multiplied by 100%, are reflected in Fig. 9.

## Discussion

When considering the MR-STAT and MRF literature, a large collection of work is available that aims at optimizing the RF pulse sequence. However, despite clear indications that the variation of phase in the RF pulses can be beneficial, almost all of the actual optimization work focuses on the amplitude of the pulses.

Here, we applied a method to optimize sequences on amplitude *and phase* of the RF pulses in several conditions. This results in the four sequences shown in the bottom row of Fig. 1, where the orange line represents the second temporal derivative of the RF phase. Note that the obtained quadratic phase coefficient is rather small: its RMS value is around 2.5°$/{T}_{R}^{2}$, which is very close to the values applied in prior work, where a manually designed ±2°$/{T}_{R}^{2}$ pattern [27, 29] was applied; it is also similar to the ±1.24°$/{T}_{R}^{2}$ pattern in Wang et al. [26], although this refers to a balanced sequence for mapping ${T}_{2}^{*}$.

Although we describe our approach as “small quadratic”, the time-variability of the “quadratic” component renders our phase-function to have all polynomial components—except the irrelevant zeroth and first order phase components. Similarly, while the quadratic components are termed “small” (in practice, on the order of 2° per ${T}_{R}^{2}$), these are not *restricted* to small values—it is an optimization result.

As shown in Fig. 6 and Table 2, the application of RF phase modulation is consistently beneficial; it brings, in average, over 30% precision gain without any cost on scan time (row (a) of Table 2). The phase modulation is particularly beneficial in SAR-constrained conditions, as shown in row (e) and—in more detail—in supplementary material 3. Similarly important is the finding in Table 2 rows (f) and (h): when applying an optimized phase modulation to the RF pulses, we see hardly any precision gain in applying inversion pulses (gain measured to be 9%), while—in absence of inversion pulses—application of a pause delivers no scan-efficiency benefit at all. This allows for contiguous sequences (i.e. sequences substantially over 10 s scan time without any pauses or magnetization-preparation), which is very relevant for applications like 3D or time-resolved acquisitions. Even when considering the common 2D approach with inversion pulse, the precision gain due to phase modulation is still substantial (around 17%), as shown by row (d) of Table 2. This suggests that phase modulation is also useful for the common 2D approach with inversion.

Collaterally, the obtained experimental results also provide insights into the correspondence between the noise levels predicted by our BLAKJac analysis and those actually measured as shown in Fig. 7. While the overall correlation between estimation and measurement is 0.88 with a slope of 0.94, per category, these values may differ: If for phantom and brain the correlation is very high (for ${T}_{1}$, 0.94 and 0.93, respectively; for ${T}_{2}$, 0.98 and 0.97 respectively), for muscle, the correlation is much lower (0.35, see red bullets); this is explainable by the rather low variability of noise levels over scenarios and conditions, which is in turn explainable by the very short ${T}_{2}$ value of muscle (approximately 25 ms): each signal is then only influenced by the most recent RF pulses, which renders slow phase-variation almost irrelevant. One can also notice that the slope is substantially lower in the bone marrow case, particularly when considering the ${T}_{2}$ maps thereof (orange bullets): a slope of 0.33 is observed, where we would ideally expect a slope of 1.0.

This suggests an additional perturbation, other than pure thermal noise such as time-dependent artefacts, e.g. motion-artefacts. In addition, BLAKJac assumes a single-component model, a perfectly controlled level of the excitation field ${B}_{1}^{+}$, a perfectly known slice-profile, absence of magnetization-transfer [47] effects etc., which also adds to some discrepancy between BLAKJac predicted ratios and actually measured ratios.

With phase, we apply more degrees of freedom (12 against 6); yet, just doubling the degrees of freedom for amplitude-only acquisitions brings only about 10% gain (not shown), as opposed to 56% when introducing phase (BLAKJac simulations on the No-pause condition).

We point out that another strategy to increase the degrees of freedom would be to vary the ${T}_{R}$ (Ref. [18]). Exploring the benefit of this strategy would go beyond the scope of this paper and we leave it to future work.

### Relationship between precision and accuracy

Intuitively, inaccuracy is increased if a method (or sequence) exhibits poor sensitivity to a parameter. In general, poor sensitivity will also lead to increased noise (i.e. decreased precision) in the reconstructed result, so one might expect that accuracy correlates with precision. Yet, there are many factors that influence accuracy, and sensitivity is just one of them. Other factors are discussed below.

### Accuracy issues in Phantom measurements

Figure 9 suggests that, in general, the Amplitude + Phase scenario leads to better accuracy than Amplitude-only, the most accurate being NoPause Amplitude + Phase. However, these findings should be interpreted with caution; the comparison to gold-standard measurements reflects a concoction of sensitivities to confounders, including B1-effects [48] and diffusion [54], whereby the sensitivity may be exemplary to a given sequence; our sequences have not been optimized on bias-minimization, which will be the subject of a separate study.

### Accuracy issues in in-vivo measurements

In-vivo, things are more complicated [47–53]. We observe that, in-vivo, the measured T1 or T2 may vary substantially over scenarios/conditions. Most notably, in the ${T}_{1}$ measured in white matter, there is a substantial difference between the inversion condition on one hand and the no-inversion conditions on the other—a sequence-to-sequence difference that is much larger than observed in phantoms.

In-vivo, the adoption of the standard Bloch equations [55] for characterizing biological tissue is clearly an over-simplification of the true system at hand and ignore effects such as magnetization transfer [47]. Furthermore, most quantitative methods, including the one presented here, implicitly assume that each voxel contains a single species—which is obviously another oversimplification.

Specifically for the white matter, we speculate that magnetization transfer is the primary mechanism behind the observed differences. The initial inversion pulse is a broadband adiabatic pulse, strongly acting on the pool of bound protons and thus inducing a substantial magnetization-transfer effect, which is substantially smaller without inversion. Speculatively, the lack of inversion may contribute to a purer in-vivo ${T}_{1}$ mapping.

An analysis of the accuracy would be particularly relevant only if the mentioned confounders, and the effect of the sequence thereupon, could be studied and optimized in isolation. This would go beyond the scope of this paper and we leave it to future research.

## Conclusion

In terms of the resulting precision, applying an optimized phase modulation to RF pulses in quantitative transient-states MRI is beneficial for almost all tested anatomies and conditions. It is particularly beneficial for SAR-restricted conditions, which is relevant for high-field systems. When applying an optimized phase modulation, the relevance of inversion pulses and pauses is substantially reduced, at least considering overall precision and in-phantom accuracy. Omission of inversion and pauses is particularly relevant for 3D or time-resolved sequences.

## Supplementary Information

Below is the link to the electronic supplementary material.

Supplementary file1 (PDF 149 KB)

Supplementary file2 (DOCX 48 KB)

Supplementary file3 (DOCX 42 KB)

## Data Availability

The referred BLAKJac code is available in Github, see 10.5281/zenodo.10072399.

## Abbreviations

BLAKJac: Block analysis of K-space view on the Jacobian
Contiguous sequence: A sequence uninterrupted by pauses or preparation pulses
MRF: Magnetic resonance fingerprinting
MR-STAT: Magnetic resonance spin tomography in the time domain
RF: Radio frequency
RF pulse sequence: The pattern of applied RF excitation pulse angles or phases, i.e. flip angle variation scheme
ROI: Region of interest
RMS: Root mean square

## References

[1] Pykett I, Mansfield P (1978) A line scan image study of a tumorous rat leg by NMR. Phys Med Biol 23:012 10.1088/0031-9155/23/5/012 715009

[2] Pedro Teixeira RA, Malik SJ, Hajnal JV (2018) Joint system relaxometry (JSR) and Cramer-Rao lower bound optimization of sequence parameters: a framework for enhanced precision of DESPOT T 1 and T 2 estimation. Magn Reson Med 79:234–245 28303617 10.1002/mrm.26670 PMC5763350

[3] Bruder H, Fischer H, Graumann R, Deimling M (1988) A new steady-state imaging sequence for simultaneous acquisition of two MR images with clearly different contrasts. Magn Reson Med 7:35–42 3386520 10.1002/mrm.1910070105

[4] Shcherbakova Y, van den Berg CAT, Moonen CTW, Bartels LW (2018) Planet: an ellipse fitting approach for simultaneous T_1_ and T_2_ mapping using phase-cycled balanced steady-state free precession. Magn Reson Med 79:711–722 28543430 10.1002/mrm.26717 PMC5811804

[5] Warntjes JBM, Dahlqvist O, Lundberg P (2007) Novel method for rapid, simultaneous T1, T2*, and proton density quantification. Magn Reson Med 57:528–537 17326183 10.1002/mrm.21165

[6] Warntjes JBM, Leinhard OD, West J, Lundberg P (2008) Rapid magnetic resonance quantification on the brain: optimization for clinical usage. Magn Reson Med 60:320–329 18666127 10.1002/mrm.21635

[7] Chen Y, Liu S, Wang Y, Kang Y, Haacke EM (2018) Strategically acquired gradient echo (STAGE) imaging, part I: creating enhanced T1 contrast and standardized susceptibility weighted imaging and quantitative susceptibility mapping. Magn Reson Imaging 46:130–139 29056394 10.1016/j.mri.2017.10.005

[8] Kvernby S, Warntjes MJB, Haraldsson H, Carlhäll C-J, Engvall J, Ebbers T (2014) Simultaneous three-dimensional myocardial T1 and T2 mapping in one breath hold with 3D-QALAS. J Cardiovasc Magn Reson 16:102 25526880 10.1186/s12968-014-0102-0 PMC4272556

[9] Ma S, Wang N, Fan Z, Kaisey M, Sicotte NL, Christodoulou AG, Li D (2021) Three-dimensional whole-brain simultaneous T1, T2, and T1 *ρ* quantification using MR multitasking: method and initial clinical experience in tissue characterization of multiple sclerosis. Magn Reson Med 85:1938–1952 33107126 10.1002/mrm.28553 PMC8244966

[10] Ma D, Gulani V, Seiberlich N, Liu K, Sunshine JL, Duerk JL, Griswold MA (2013) Magnetic resonance fingerprinting. Nature 495:187–192 23486058 10.1038/nature11971 PMC3602925

[11] Sbrizzi A, van der Heide O, Cloos M, van der Toorn A, Hoogduin H, Luijten PR, van den Berg CAT (2018) Fast quantitative MRI as a nonlinear tomography problem. Magn Reson Imaging 46:56–63 29103975 10.1016/j.mri.2017.10.015 PMC6080622

[12] Gómez PA, Molina-Romero M, Buonincontri G, Menzel MI, Menze BH (2019) Designing contrasts for rapid, simultaneous parameter quantification and flow visualization with quantitative transient-state imaging. Sci Rep. 10.1038/s41598-019-44832-w 31186480 10.1038/s41598-019-44832-w PMC6560213

[13] Assländer J, Novikov DS, Lattanzi R, Sodickson DK, Cloos MA (2019) Hybrid-state free precession in nuclear magnetic resonance. Commun Phys 2:1–12 10.1038/s42005-019-0174-0 PMC6641569 31328174

[14] Zhao B, Haldar JP, Setsompop K, Wald LL (2016) Optimal experiment design for magnetic resonance fingerprinting. 2016 38th Annual international conference of the IEEE engineering in medicine and biology society (EMBC). IEEE, pp 453–456 10.1109/EMBC.2016.7590737 PMC5464426 28268369

[15] Sommer K, Amthor T, Doneva M, Koken P, Meineke J, Börnert P (2017) Towards predicting the encoding capability of MR fingerprinting sequences. Magn Reson Imaging 41:7–14 28684268 10.1016/j.mri.2017.06.015

[16] Cohen O, Rosen MS (2017) Algorithm comparison for schedule optimization in MR fingerprinting. Magn Reson Imaging 41:15–21 28238942 10.1016/j.mri.2017.02.010 PMC10326798

[17] Stolk CC, Sbrizzi A (2019) Understanding the combined effect of k-space undersampling and transient states excitation in MR fingerprinting reconstructions. IEEE Trans Med Imaging 38:2445–2455 30802852 10.1109/TMI.2019.2900585

[18] Lee PK, Watkins LE, Anderson TI, Buonincontri G, Hargreaves BA (2019) Flexible and efficient optimization of quantitative sequences using automatic differentiation of Bloch simulations. Magn Reson Med 82:1438–1451 31131500 10.1002/mrm.27832 PMC8057531

[19] Leitão D, Teixeira RPAG, Price A, Uus A, Hajnal JV, Malik SJ (2021) Efficiency analysis for quantitative MRI of T1 and T2 relaxometry methods. Phys Med Biol 66:15NT02 10.1088/1361-6560/ac101f PMC8312556 34192676

[20] Jordan SP, Hu S, Rozada I, McGivney DF, Boyacioğlu R, Jacob DC, Huang S, Beverland M, Katzgraber HG, Troyer M et al (2021) Automated design of pulse sequences for magnetic resonance fingerprinting using physics-inspired optimization. Proc Natl Acad Sci U S A 118:e2020516118 34593630 10.1073/pnas.2020516118 PMC8501900

[21] Assländer J (2021) A perspective on MR fingerprinting. J Magn Reson Imaging 53:676–685 32286717 10.1002/jmri.27134 PMC7554187

[22] Mickevicius NJ, Nencka AS, Paulson ES (2020) Reducing the dimensionality of optimal experiment design for magnetic resonance fingerprinting. Preprint at 10.48550/arXiv.2010.00674

[23] Hu S, McGivney D, Griswold M, Ma D (2022) Optimal experimental design of MR Fingerprinting for simultaneous quantification of T1, T2, and ADC. ISMRM. p 4802

[24] Fuderer M, Van Der Heide O, Liu H, Van Den Berg CAT, Sbrizzi A (2023) Efficient performance analysis and optimization of transient-state sequences for multiparametric magnetic resonance imaging. NMR Biomed. 10.1002/nbm.4864 36321222 10.1002/nbm.4864 PMC10078474

[25] Heesterbeek DG, Koolstra K, van Osch MJ, van Gijzen MB, Vos FM, Nagtegaal MA, David Heesterbeek CG (2022) Mitigating undersampling errors in MR fingerprinting by sequence optimization. Magn Reson Med. 10.1002/mrm.29554 36458688 10.1002/mrm.29554

[26] Wang CY, Coppo S, Mehta BB, Seiberlich N, Yu X, Griswold MA (2019) Magnetic resonance fingerprinting with quadratic RF phase for measurement of T2* simultaneously with δf, T1, and T2. Magn Reson Med 81:1849 30499221 10.1002/mrm.27543 PMC7325599

[27] Wang X, Hernando D, Reeder SB (2020) Phase-based T2 mapping with gradient echo imaging. Magn Reson Med 84:609–619 31872470 10.1002/mrm.28138 PMC7180093

[28] Boyacioglu R, Wang C, Ma D, McGivney DF, Yu X, Griswold MA (2021) 3D magnetic resonance fingerprinting with quadratic RF phase. Magn Reson Med 85:2084–2094 33179822 10.1002/mrm.28581 PMC12988812

[29] Liu H, Bruijnen T, van Haandel M, van der Heide O, Fuderer M, van den Berg CAT, Sbrizzi A (2022) Increasing the T2 sensitivity of MR-STAT sequences by small quadratic RF phase increments. Proceedings of the ISMRM. p 0625

[30] Zhao B, Setsompop K, Ye H, Cauley SF, Wald LL (2016) Maximum likelihood reconstruction for magnetic resonance fingerprinting. IEEE Trans Med Imaging 35:1812–1823 26915119 10.1109/TMI.2016.2531640 PMC5271418

[31] Fuderer M, van der Heide O, Liu H, van den Berg CAT, Sbrizzi A (2022) Non-steady-state sequences for multi-parametric MRI need to be evaluated in the context of gradient-encoding. ISMRM. p 2786

[32] Yu Z, Zhao T, Assländer J, Lattanzi R, Sodickson DK, Cloos MA (2018) Exploring the sensitivity of magnetic resonance fingerprinting to motion. Magn Reson Imaging 54:241–248 30193953 10.1016/j.mri.2018.09.002 PMC6215476

[33] Amthor T, Doneva M, Koken P, Sommer K, Meineke J, Börnert P (2017) Magnetic resonance fingerprinting with short relaxation intervals. Magn Reson Imaging 41:22–28 28666939 10.1016/j.mri.2017.06.014

[34] Liao C, Bilgic B, Manhard MK, Zhao B, Cao X, Zhong J, Wald LL, Setsompop K (2017) 3D MR fingerprinting with accelerated stack-of-spirals and hybrid sliding-window and GRAPPA reconstruction. Neuroimage 162:13–22 28842384 10.1016/j.neuroimage.2017.08.030 PMC6031129

[35] Liu H, Van Der Heide O, Versteeg E, Froeling M, Fuderer M, Xu F, Van Den Berg CAT, Sbrizzi A (2023) A three-dimensional magnetic resonance spin tomography in time-domain protocol for high-resolution multiparametric quantitative magnetic resonance imaging. NMR Biomed. 10.1002/nbm.5050 37857335 10.1002/nbm.5050

[36] Li T, Cui D, Ren G, Hui ES, Cai J (2021) Investigation of the effect of acquisition schemes on time-resolved magnetic resonance fingerprinting. Phys Med Biol 66:095013 10.1088/1361-6560/abf51f 33823496

[37] Flassbeck S, Schmidt S, Bachert P, Ladd ME, Schmitter S (2019) Flow MR fingerprinting. Magn Reson Med 81:2536–2550 30506796 10.1002/mrm.27588

[38] Zur Y, Wood ML, Neuringer LJ (1991) Spoiling of transverse magnetization in steady-state sequences. Magn Reson Med 21:251–263 1745124 10.1002/mrm.1910210210

[39] Cloos MA, Knoll F, Zhao T, Block KT, Bruno M, Wiggins GC, Sodickson DK (2016) Multiparametric imaging with heterogeneous radiofrequency fields. Nat Commun 7:12445 27526996 10.1038/ncomms12445 PMC4990694

[40] Wyatt CR, Barbara TM, Guimaraes AR (2020) T1ρ magnetic resonance fingerprinting. NMR Biomed. 10.1002/nbm.4284 32125050 10.1002/nbm.4284 PMC8818303

[41] Weigel M (2015) Extended phase graphs: dephasing, RF pulses, and echoes-pure and simple. J Magn Reson Imaging 41:266–295 24737382 10.1002/jmri.24619

[42] Fuderer M (2023) Resources for BLAKJac sequence optimization. 10.5281/zenodo.10072399

[43] Nehrke K, Boernert P (2012) DREAM-a novel approach for robust, ultrafast, multislice B 1 mapping. Magn Reson Med 68:1517–1526 22252850 10.1002/mrm.24158

[44] Diagnostic Sonar Ltd (1995) Eurospin II magnetic resonance quality assessment test object 5

[45] Shaffer JP (1992) Caution on the use of variance ratios: a comment. Rev Educ Res 62:429

[46] Hedges Lv, Gurevitch J, Curtis PS (1999) The meta-analysis of response ratios in experimental ecology. Ecology 80:1150

[47] Wolff SD, Balaban RS (1989) Magnetization transfer contrast (MTC) and tissue water proton relaxation *in vivo*. Magn Reson Med 10:135–144 2547135 10.1002/mrm.1910100113

[48] Hamilton JI, Jiang Y, Ma D, Lo WC, Gulani V, Griswold M, Seiberlich N (2018) Investigating and reducing the effects of confounding factors for robust T1 and T2 mapping with cardiac MR fingerprinting. Magn Reson Imaging 53:40–51 29964183 10.1016/j.mri.2018.06.018 PMC7755105

[49] Stikov N, Boudreau M, Levesque IR, Tardif CL, Barral JK, Pike GB (2015) On the accuracy of T _1_ mapping: searching for common ground. Magn Reson Med 73:514–522 24578189 10.1002/mrm.25135

[50] Bojorquez JZ, Bricq S, Acquitter C, Brunotte F, Walker PM, Lalande A (2017) What are normal relaxation times of tissues at 3 T? Magn Reson Imaging 35:69–80 27594531 10.1016/j.mri.2016.08.021

[51] Pai A, Li X, Majumdar S (2008) A comparative study at 3 T of sequence dependence of T2 quantitation in the knee. Magn Reson Imaging 26:1215–1220 18502073 10.1016/j.mri.2008.02.017 PMC2849643

[52] Matzat SJ, McWalter EJ, Kogan F, Chen W, Gold GE (2015) T _2_ relaxation time quantitation differs between pulse sequences in articular cartilage. J Magn Reson Imaging 42:105–113 25244647 10.1002/jmri.24757 PMC4369475

[53] Wright PJ, Mougin OE, Totman JJ, Peters AM, Brookes MJ, Coxon R, Morris PE, Clemence M, Francis ST, Bowtell RW et al (2008) Water proton T 1 measurements in brain tissue at 7, 3, and 1.5 T using IR-EPI, IR-TSE, and MPRAGE: results and optimization. Magn Reson Mater Phy 21:121–130 10.1007/s10334-008-0104-8 18259791

[54] Fuderer M, Van Der Heide O, Liu H, Van Den Berg CAT, Sbrizzi A (2024) Water diffusion and T 2 quantification in transient-state MRI: the effect of RF pulse sequence. NMR Biomed. 10.1002/nbm.5044 37772434 10.1002/nbm.5044

[55] Bloch F (1946) Nuclear induction. Phys Rev 70:460–474

[56] Chen Y, Panda A, Pahwa S, Hamilton JI, Dastmalchian S, McGivney DF, Ma D, Batesole J, Seiberlich N, Griswold MA, Plecha D, Gulani V (2019) Three-dimensional MR fingerprinting for quantitative breast imaging. Radiology 290:33–40 30375925 10.1148/radiol.2018180836 PMC6312432

