# Supplementary material for: RF phase modulation improves quantitative transient state sequences under constrained conditions
Source: MAGMA. 2025 Sep 11;39(1):47–59. doi: 10.1007/s10334-025-01293-9 (PMC12901244; doi:10.1007/s10334-025-01293-9)
Supplement: Supplementary file 1 — Supplementary file1 (PDF 149 KB) [file 10334_2025_1293_MOESM1_ESM.pdf]

Details of BLAKJac choices

For all 8 combinations of scenarios and conditions, the optimization was aimed at minimizing the maximum relative noise level in the T_1 and T_2 maps, averaged over 7 different reference (T_1, T_2) pairs listed in the table below

T_1	T_2
0.82s	0.051s
0.67s	0.21s
1.22s	0.125s
0.25s	0.046s
2.2s	0.31s
0.37s	0.046s
0.37s	0.125s

These pairs span the set of frequently occurring human tissues as well as the phantom vials. In mathematical notation: the criterion was set to minimize $\max\left(\frac{1}{7}\sum_{i=1}^{7}\frac{\sigma_{T1,i}}{T_{1,\mathrm{ref}_i}}, \frac{1}{7}\sum_{i=1}^{7}\frac{\sigma_{T2,i}}{T_{2,\mathrm{ref}_i}}\right)$, where $\sigma_{T1,i}$ is the Cramer-Rao Bound estimation of the noise in the T_1-map for $(T_1, T_2) \approx (T_{1,\mathrm{ref}_i}, T_{2,\mathrm{ref}_i})$. The Cramer-Rao Bound of the sequence was efficiently calculated by the BLAKJac model. For each of the 8 combinations of scenario × condition, the optimization was run 6 times with different random initialization patterns; the relative performance difference between the 6 resulting sequences was small (<10%), and the best performing of the 6 was selected.
