# Supplementary material for: RF phase modulation improves quantitative transient state sequences under constrained conditions
Source: MAGMA. 2025 Sep 11;39(1):47–59. doi: 10.1007/s10334-025-01293-9 (PMC12901244; doi:10.1007/s10334-025-01293-9)

**Influence of the choice of the pause-length**

In the main article, only two values of the pause were considered: 0s (the No-Pause condition) or 5s (all other conditions).

In this supporting material, we examine the effects of other choices of the pause. This is enabled by BLAKJac simulations, which allow to examine the effect of a variety of pause-lengths. This is evaluated here for the condition without inversion pulses (i.e. the Base condition, but with variable pause lengths). The bullets in Figure S-5 shows the noise standard deviation (arbitrary units) for pauses between 0s and 10s, for Amplitude-only and for Amplitude+Phase. The figure suggests that, for longer pauses, the noise level decreases (i.e. the SNR increases) with increasing pause length, although the difference is very minor (<3%) between 5s and 10s.

The crosses reflect the noise level normalized for scan efficiency; these have been obtained by multiplying the noise levels by $\sqrt{\frac{T_{S}+T_{P}}{T_{S}}}$, where $T_{S}$ is the scan time of a segment (in our case, $T_{s}=13.44\text{s}$) and $T_{P}$ is the length of the pause. The orange crosses indicate that, in terms of scan efficiency, the length of a pause is irrelevant in the Amplitude+Phase scenario (irrelevant within $\pm3\%$, when ignoring the cross at 10s). For Amplitude-only, the no-pause sequence has a slight higher efficiency (around 10%) over sequence with nonzero pauses.


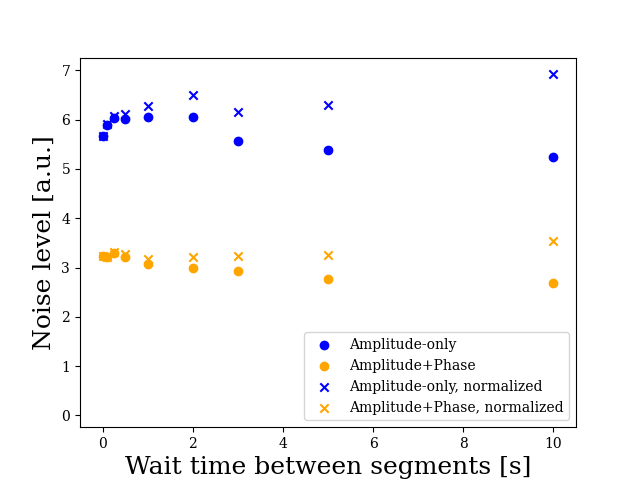

Supplement: Supplementary file 2 — Supplementary file2 (DOCX 48 KB) [file 10334_2025_1293_MOESM2_ESM.docx]
