# Supplementary material for: RF phase modulation improves quantitative transient state sequences under constrained conditions
Source: MAGMA. 2025 Sep 11;39(1):47–59. doi: 10.1007/s10334-025-01293-9 (PMC12901244; doi:10.1007/s10334-025-01293-9)

**Influence of allowed RF power on SNR**

Noise levels for BLAKJac-optimized sequences have been calculated for a variety of Root Mean Square (RMS) values of the flip angles. This is shown in Figure S-6. As expected, the noise level of the reconstructed $T_{1}$ and $T_{2}$ maps decreases (i.e. the SNR increases) with the increase of allowed RMS value of the RF excitation angles. For the Amplitude+Phase scenario (orange bullets), the reconstruction noise levels off around an allowed excitation angle of 40 degrees: the optimized RMS excitation angle never exceeds 36 degrees even if more is allowed (details not shown). As a consequence, the noise level does not decrease beyond an allowed angle of 40 degrees. The Amplitude-only scenario (blue bullets) is much more SAR demanding and shows very high noise levels when stringent restrictions on the RMS excitation angles are imposed.


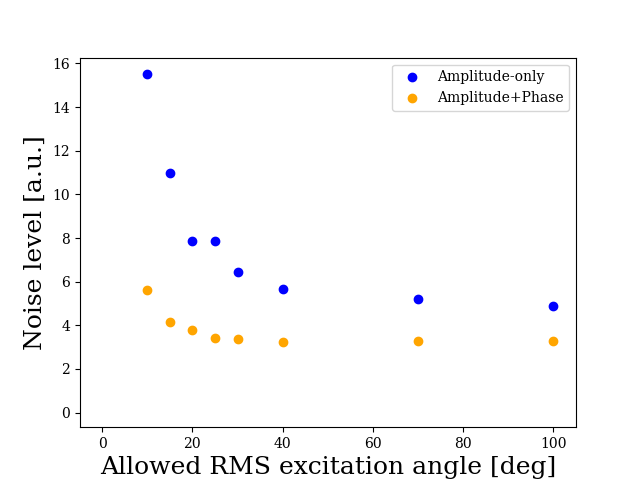

Supplement: Supplementary file 3 — Supplementary file3 (DOCX 42 KB) [file 10334_2025_1293_MOESM3_ESM.docx]
